# A Participatory Agent-Based Simulation for Indoor Evacuation Supported by Google Glass

**DOI:** 10.3390/s16091360

**Published:** 2016-08-24

**Authors:** Jesús M. Sánchez, Álvaro Carrera, Carlos Á. Iglesias, Emilio Serrano

**Affiliations:** 1 Departamento de Ingeniería de Sistemas Telemáticos, Escuela Técnica Superior de Ingenieros de Telecomunicación, Universidad Politécnica de Madrid, 28040 Madrid, Spain; jesusmanuel.sanchez.martinez@alumnos.upm.es (J.M.S.); cif@dit.upm.es (C.Á.I.); 2 Departamento de Inteligencia Artificial, Escuela Técnica Superior de Ingenieros Informáticos, Universidad Politécnica de Madrid, Boadilla del Monte, 28660 Madrid, Spain; emilioserra@fi.upm.es

**Keywords:** ambient intelligence, agent-based social simulation, participatory simulation, emergency plan, routing, indoor mapping, Representational state transfer (REST)

## Abstract

Indoor evacuation systems are needed for rescue and safety management. One of the challenges is to provide users with personalized evacuation routes in real time. To this end, this project aims at exploring the possibilities of Google Glass technology for participatory multiagent indoor evacuation simulations. Participatory multiagent simulation combines scenario-guided agents and humans equipped with Google Glass that coexist in a shared virtual space and jointly perform simulations. The paper proposes an architecture for participatory multiagent simulation in order to combine devices (Google Glass and/or smartphones) with an agent-based social simulator and indoor tracking services.

## 1. Introduction

Recent tragedies involving crowd evacuations in events, such as massive parties, terrorist attacks and sports events, have focused attention on the importance of effective evacuations. Emergency evacuation, known as egress, is a critical component of emergency response and requires developing in advance evacuation preparation activities that ensure that people can get to safety in case of emergency. In order to define effective evacuation plans, understanding disasters and crowd emergency evacuation behavior is essential [1].

With the aim of understanding crowd behavior in emergencies, mass psychology has developed a number of theories, summarized in Section 2. Some of its findings are that real-time information and communication are critical aspects in preventing crowd disasters [2]. Public address systems and video screens are usually used for communicating with the crowd about the emergency and for providing evacuation instructions. Nevertheless, these systems are not capable of providing individualized evacuation instructions to suit user’s characteristics. For this purpose, smartphones [3,4] and tablets [5] are popular options. Moreover, several experiences have explored the use of wearable devices, such as eye-tracking glasses [6,7], for providing instructions.

As full-scale evacuation demonstration is not viable because of ethical, practical and financial issues [8], models and simulations of crowd behavior in simulations are widely used to analyze the effectiveness of evacuation preparation activities. Different computer-based simulation approaches are used in the literature for evacuation, such as flow dynamics [9] or cellular automata [10,11]. However, agent-based simulation (ABS) has been used as the preferred method to simulate crowd behavior [12], because agents are particularly suitable for modeling human behavior [13], as its characteristics can be objectively mapped to agent behavior. In order to evaluate how users’ communication is improved, we use participatory agent-based simulations [14], which are agent-based simulations where some users are included as human-controlled avatars. Given that integration of agent-based social simulators with external applications has received little attention by the research community, we propose a general framework for this purpose that is used in the design of the overall solution.

In this work, we explore the use of a new device, Google Glass [15], for evacuation purposes in order to improve the communication of personalized evacuation routes using different sensing mechanisms for indoor positioning and tracking. Google Glass is a wearable headset computer that includes an optical head-mounted display. It was selected for this evacuation purpose to provide a hand-free tool, which provides faster and safer ways of showing information to the user than others, like smartphones and tablets, as confirmed during the experimentation.

The rest of this paper is organized as follows. Firstly, in Section 2, we discuss some related works in the field of social theories about crowd behavior in evacuations and the different approaches of agent-based participatory simulations. Section 3 presents how an existing agent-based social simulator can be extended for participatory simulation based on REST principles. Later, Section 4 describes the architecture of a participatory agent-based simulation integrated with Google Glass devices, which is validated in a real-life evacuation scenario in Section 5. Finally, Section 6 shows the main conclusions of the paper and provides an outlook on future work.

## 2. Background

This section describes the background and related work for the architecture proposed in this paper. First, in Section 2.1, we review social theories about crowd behavior in evacuations that highlight the importance of communication in evacuation emergencies. Then, we review existing experiences in participatory agent-based simulations with a focus on their architecture for integrating human agents and the agent-based simulator in Section 2.2.

### 2.1. Social Theories about Crowd Behavior in Evacuations

Existing research of social theories has explored crowd behaviors [16] in emergencies from the following perspectives: decision making, exit times, clinical issues and crowd behavior [1,12].

Firstly, exit selection and the time it takes to decide to evacuate [12,17] have a great influence on the outcome of an evacuation, and this is considered a complex decision process. People are influenced by aspects, such as their familiarity with exits or their visibility [18]. Other relevant aspects for exit selection come from the interaction of people with other users, where relevant aspects are their personality (e.g., shy) to follow or cooperate with other users [19,20].

Secondly, some researchers have analyzed clinical issues, such as freezing or becoming disassociated from reality, which are also potentially dangerous [12]. However, those researchers found other interesting findings; around 50% of emergency survivors referred unambiguously to a sense of unity or togetherness with the rest of the crowd during the emergency.

Summarizing, several models and theories have been developed for understanding crowd behavior [16,21,22]. While the traditional model of mass panic or irrational people has been observed in very few cases [23], the affiliation model of evacuation behavior explains why family groups often escape or die together [24]. The normative approach explains that the same social conventions are followed in emergency situations [25], e.g., people help the elderly. Other approaches like social identity help to explain collective behaviors among unfamiliar crowd members during emergencies [26].

As a result of this better understanding of crowd behavior, several key factors have been identified for improving evacuation processes [1]: interpretation, preparation and action. The interpretation of the information provided about the emergency is vital to improve evacuation efficiency. Thus, communication is essential and should be prioritized. Preparation refers to training activities for both users and leader figures. Finally, action refers to the behavior during the evacuation and how users accept that they are in an emergency situation, abandon their habits and follow the provided evacuation instructions.

Based on these concepts, a number of domain-specific simulators deal with evacuations of indoor environments for emergencies [27]. Among others, we can find some examples in EVACNET [28], STEPS [29], SimWalk [30] or WAYOUT [31]. These are excellent tools for emergency modeling, but they do not consider some interesting features for intelligent real-time evacuation planning, such as the sensor model and/or actuators. Moreover, they tend to ignore the multi-agent paradigm, which is an intuitive manner of studying complex adaptive systems; and more importantly, the source code is often not available; and thus, researchers cannot extend, reuse or simply learn from them.

### 2.2. Agent-Based Participatory Simulations

Computer-based simulations have become a useful part of modeling many natural and human systems over the last few years [32]. Among the different existing types of simulations, the use of agent-based social simulation (ABSS) is one of the most representative research streams. This type of simulation is a computer-assisted simulation technique used to model artificial societies populated with multiple autonomous entities, called agents, which act autonomously by employing some knowledge for validating some hypotheses [33]. However, in recent years, a new trend is emerging for ABS, the agent-based participatory simulations. Participatory modeling aims to improve the design of agent-based simulation models with the participation of the stakeholders [34,35,36]. Two main approaches are followed: (i) participatory simulations in a virtual world [37], where agents and human-controlled avatars coexist in a shared virtual space; and (ii) participatory simulations in an augmented real world [14], where people and virtual agents coexist in an augmented real space. Moreover, these two approaches can be combined developing the simulation in a virtual space as a previous experiment to prepare the simulation in the augmented real space [14].

Several authors have developed participatory simulations in a virtual world, using role playing games [34,38]. The integration of the agent-based simulator and human agents is carried out through an interface where users are asked to fill out forms and the collected data that feeds the agent-based model. Murakami et al. [39] propose to develop a platform, FreeWalk/Q [40], for participatory simulation that provides a 3D virtual space where agents and avatars can interact. The platform enables agents and people to interact with each other based on the same interaction model. While agents are controlled through the platform’s APIs, human participants enter in the space as avatars that are controlled through UI devices connected to the platform.

One of the most relevant works in the development of participatory simulations in the real world (also known as augmented experiment [14]) is the work by Nakajima et al. [3,41], where the multiagent platform Caribbean/Q is developed for providing a large-scale simulation where a guiding agent is assigned to each human. In this platform, an interaction protocol interpreter is designed to control agent behavior, so that agent development is decoupled from the protocol description. Nevertheless, the interaction of the platform with mobile phones is not detailed.

## 3. RESTful Participatory Agent-Based Simulation Architecture

As discussed previously, the definition of an architecture for integrating real users with an agent-based simulator is worth examining for its own sake. In this section, we propose to adopt a RESTful architecture to this end.

Representational state transfer (REST) [42] is a network-based architectural style that has been used to guide the design and development of the architecture for the modern web. The main concepts of REST are resource, representation and state. Resources are any information that can be named and uniquely identified by an identifier. Servers expose resources that can be consulted by clients through connectors and transfer resource representations. Client-server interaction are stateless, so each request should contain all of the information necessary for a connector to understand the request. While servers should manage resource state, clients are responsible for managing the application state. The RESTful protocol is commonly used over HTTP. It uses a set of standardized verbs (GET, PUT, POST, DELETE) to retrieve, create, update or delete resources that are identified by Uniform Resource Identifiers (URI). Resources can have several representation formats, such as JSON, XML or HTML.

Regarding agent-based simulators, integrating services and event external data (e.g., Geographic Information Systems (GIS)) is a difficult process [43]. Several works have proposed different mechanisms for integrating intelligent agents and web services, as surveyed in [44]. Existing solutions provide mappings between addressing and messaging schemes in web services and agent systems. A gateway publishes web service descriptions into FIPA’s directory facilitator and vice versa. Some of the most common frameworks for agent-based modeling, such as Netlogo [45], Repast [46], GAMA [47] or MASON [48]), are now able to integrate GIS.

**Figure 1:**
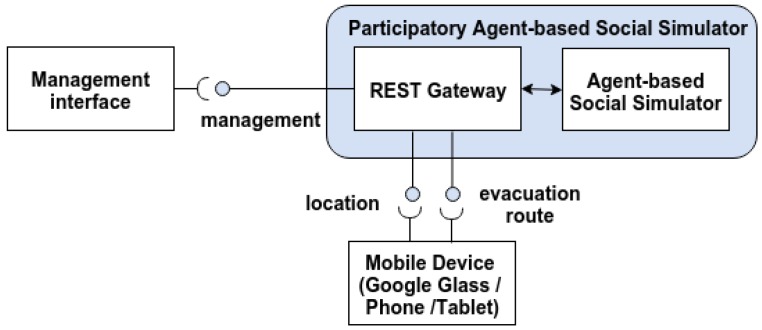
RESTful architecture for participatory agent-based simulation.

We propose to follow a RESTful architecture for participatory agent-based simulators (Figure 1), which can be used both in shared virtual spaces and real scenarios. The architecture consists of three modules: the participatory agent simulator, mobile devices and a management interface.

The participatory agent simulator is an agent-based simulator that is enhanced by a RESTful gateway, which exposes relevant parts of the agent system as resources. The RESTful gateway follows a front controller design pattern that handles all of the requests and, then, dispatches them to the appropriate handler in the agent architecture. A detailed explanation of the REST API is described below. Both Google Glass and mobile devices interact with the social simulator through the REST API for providing their location and receiving information about the position of the rest of the agents, in order to represent them in an augmented user interface.

During the simulation, users are included in the virtual world as another agent, and their location in the simulation is updated continuously through POST messages sent to the RESTful gateway. In addition, they can receive guiding instructions for evacuation. Those instructions can change during the simulated evacuation based on user’s localization, since an exit can collapse or the emergency (for example, a fire) can block a route that initially was accessible.

Finally, the management interface provides the capability to define scenarios and manage the simulation execution through a web interface. It is an optional element, since these operations can be usually done through the management interface of the agent simulation system, although this enables seamless access to management functionalities from mobile or web user interfaces. An implementation of this approach has been done with the agent-based simulator UbikSim 2.0 (License GPLv3, GSI, UPM, Madrid, Spain) [49].

**Figure 2:**
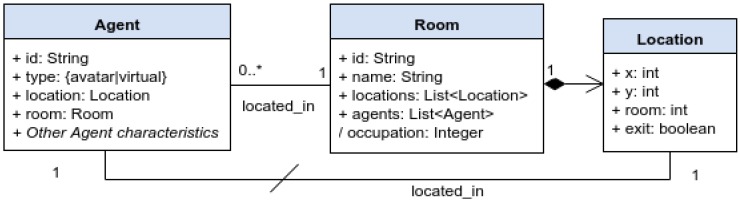
Domain model of the evacuation scenario.

**Figure 3:**
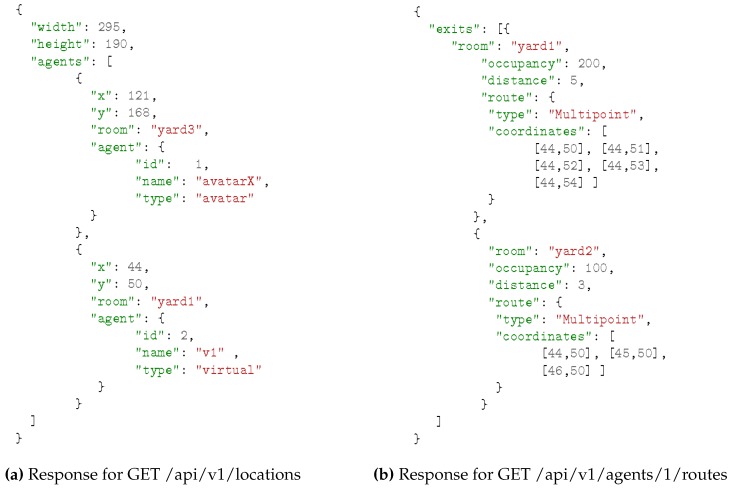
Examples of the JSON responses.

The design of RESTful services accessed by mobile devices depends on the problem we are addressing. In our evacuation scenario, we will follow RESTful design best practices [50,51] for defining them. First, resources are identified, as shown in Figure 2. In our scenario, agents (virtual or avatars) are located in rooms. The space is represented as a grid, with a discrete number of locations. Therefore, each room contains several locations, and walls are considered as non-accessible for users and/or agents. Moreover, some of those locations are exits of the room (i.e., doors). Then, resources are named with URIs and mapped to endpoints, as illustrated in Table 1, where API versioning has been followed and resources have been linked to improve its usability. Finally, resources have been represented with JSON as shown in Figure 3, following GeoJSON conventions for the routes.

## 4. Developing a Google Glass-Enabled Evacuation System Based on the RESTful Participatory Architecture

The main challenges for developing the evacuation system are: (i) to obtain accurate indoor localization of real users; (ii) to visualize the participatory simulation in Google Glass; and (iii) to provide personalized evacuation instructions in the Google Glass UI.

**Figure 4:**
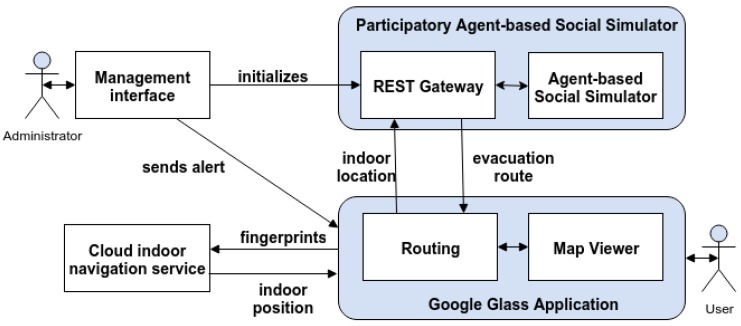
Architecture of the participatory agent-based system for Google Glass.

In order to meet these goals, the RESTful participatory architecture introduced previously has been extended as depicted in Figure 4. The components are described in the subsections below.

### 4.1. Management Interface

**Figure 5:**
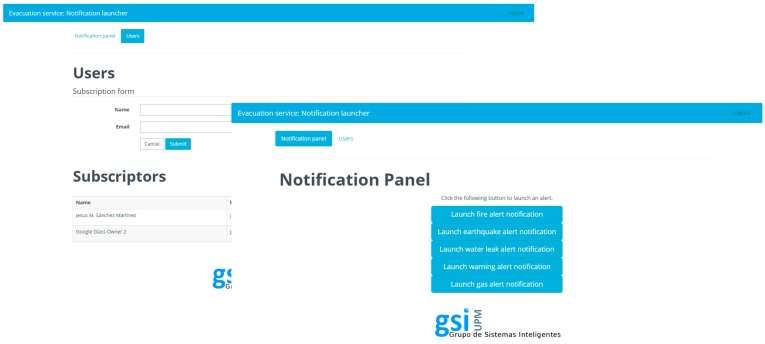
Dashboard.

A web application for management has been developed so that an administrator can carry out three functions: user management and authorization; emergency notification and emergency simulation management. Several screenshots are shown in Figure 5.

User management and authorization: Google Glass technology requires users to authorize external services to send them notifications using OAuth 2.0 credentials (IETF RFC6749, Microsoft, https://tools.ietf.org/html/rfc6749). The administration interface provides a facility to register users and request their authorization, so that they can be notified in case of emergency. The Google Glass Evacuation application uses the Google Mirror API [15] to be able to receive notifications. This API works as an intermediary between our server and the Google Glass. When users load the application for the first time, they are asked to grant permission for the application to access their Google account that has been linked with the Google Glass device. Once users have granted the application, they can receive notifications from the administrator in their timeline.

Simulation management: In addition, at the same time that the administrator controls the notifications, the corresponding actions are requested for the agent-based simulator through the RESTful management interface. The actions that have been implemented are /api/v1/control/play to create a random emergency and actions for controlling the execution of the simulation (play, pause and stop).

**Figure 6:**
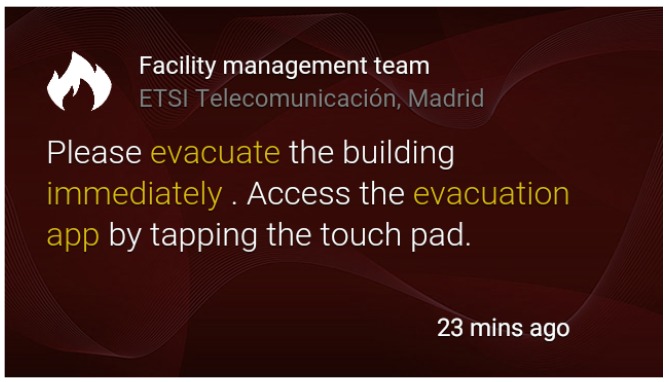
Fire card.

**Figure 7:**
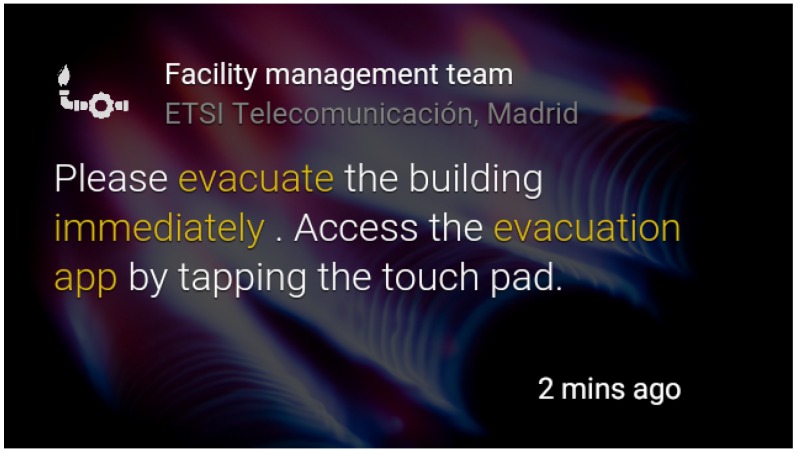
Gas leak card.

Emergency notification: The management web application provides an effective way to send users notifications about the simulated emergency. For this purpose, static cards [15] have been used. Five types of cards have been designed depending on the type of emergency: fire, earthquake, water leak, gas leak and warning. These cards are written in HTML, a language that Google Glass can read and represent on the screen, as exemplified in Figure 6 and Figure 7. They count with the following built-in actions:

- Read aloud: By accessing this action, the user is able to hear the “evacuation alert” warning him or her about the kind of notification.
- Delete: The user is also capable of deleting the card from the timeline if he or she wants to.
- Open website: This is the most interesting action. It is what we use to connect the alert to the GDK (Google Glass Development Kit) application. By accessing this action, the user will execute a customized url (android scheme) opening the evacuation app, presented in Section 4.3.

### 4.2. UbikSim Agent-Based Social Simulator

An adapted version of the agent-based social simulator UbikSim 2.0 [49] is used to recreate the human behavior inside a building. For that purpose, the map of the building where the emergency simulation takes place is modeled and represented on this tool. UbikSim is a framework used to develop social simulation, which emphasizes the modeling of realistic indoor environments and human behaviors and the evaluation of ubiquitous computing and ambient intelligence systems. UbikSim is written in Java and employs a number of third-party libraries, such as SweetHome3D and MASON.

UbikSim is responsible for simulating virtual users under the emergency, as well as calculating exit routes for the human participants according to different criteria (e.g., least crowded exit, closest exit, etc.). For this work, UbikSim has been extended, and a web gateway has been implemented to enable participatory simulation following the architecture described previously in Section 3.

**Figure 8:**
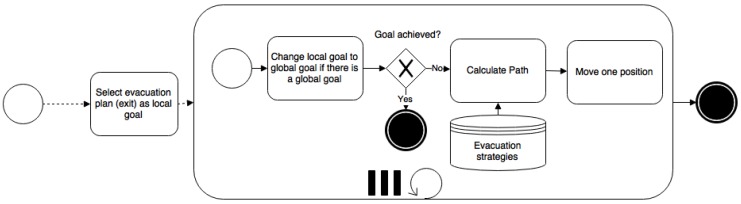
Agent behavior in UbikSim 2.0 [49].

Agents in the simulation follow an evacuation route navigating through the map. That navigation is based on a cell grid. Each cell in the environment is considered a graph node; A* [52] is applied to detect a destiny; agents follow the path; and A* is executed again if some mobile objects or hazards are in the middle of the path to avoid them. This always gets a path if there is one. However, it is computationally costly, especially when the cell size is very small. On the other hand, the time needed is very reasonable since A* uses heuristic algorithms to avoid exploring all of the grid/graph. Moreover, the time from agents’ view point is not affected if the pathfinding takes a few extra seconds since it is the “time step” that makes them age. An alternative is to use navigation meshes. However, this involves including an extra layer of complexity to build the mesh and deciding what to do when nodes in the mesh are not reachable. Another option is the use of more advanced algorithms, such as D* lite [53], which does not have to repeat all operations to find a path when a mobile obstacle is detected. However, this involves using a lot of memory to store network information for each agent, so it is not usually employed in video games and simulations where there are a large number of mobile agents. This agent behavior is shown in Figure 8.

Furthermore, UbikSim 2.0 [49] implements several evacuation plans as summarized below.

1. Random initial wait: This policy consists of making agents wait a random number of simulation steps (zero to 50) before starting the flight to minimize breakdowns in access to exits. As explained, by default, the service provides users with the shortest path to the closest exit.
2. Distributing exits based on escape order: The first user evacuated receives a path to the first exit, the second to the second and the third to the third, whereas the fourth user receives the first exit again (assuming three exits, as in the environment modeled), and so on.
3. Avoiding the exit closest to the fire: After calculating the Euclidean distance to the fire (specifically, to the position of the fire calculated based on the sensors excited), the closest stairway to that position is discarded for the evacuation.
4. Avoiding routes that include positions with fire: To improve the plan shown above, only routes that include positions with fire are ruled out. Therefore, if it is possible, the service makes use of all exits, and if more than one fire escape is risky, they are not used.
5. Putting out the fire: A random user is chosen as the emergency manager and led to the lobby to activate sprinklers that put out the fire. Then, the service guides this user to the closest fire escape.

### 4.3. Google Glass Application

**Figure 9:**
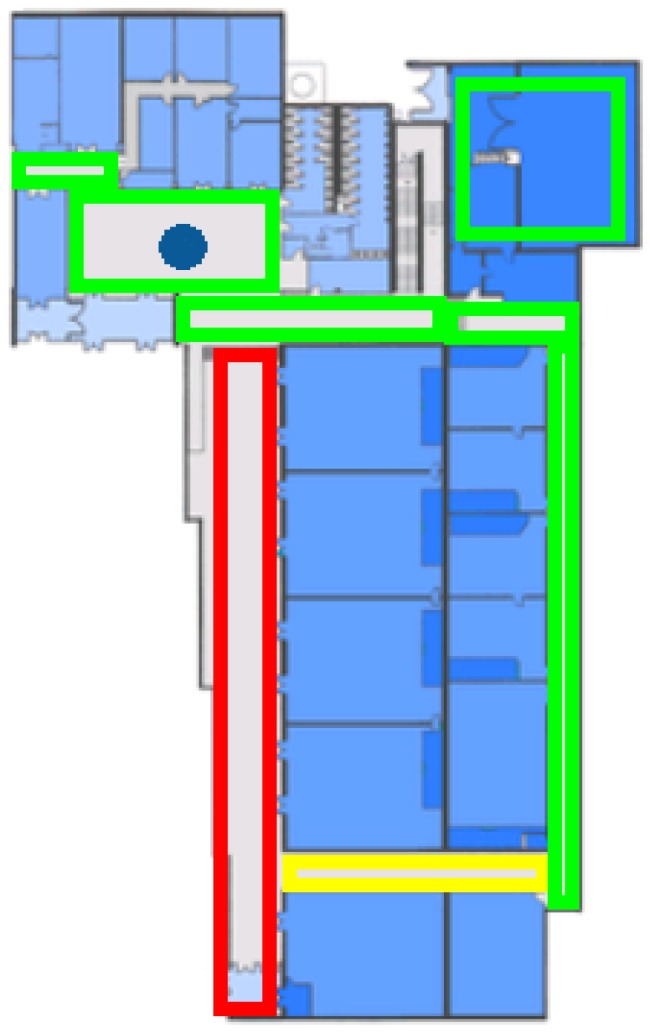
Google Glass app screen.

The developed Google Glass application provides a visualization of the building footprint to the user, where his or her location is pointed at with a circle, and the chosen evacuation route towards an exit is shown in blue. The map also provides a room congestion indicator based on colored rectangles (red means highly crowded; green means little congestion), as shown in Figure 9.

The evacuation route is sent by the social simulator REST gateway (e.g., /api/v1/agents/1/routes), and its visualization is based on the indoor localization provided by the INDOO.RS indoor localization system [54]. INDOO.RS provides several methods for providing accurate indoor localization, such as WiFi fingerprinting techniques and proximity beacons. Once the application is launched, it sends a request to the INDOO.RS server to obtain the building map providing the API key and the building ID. The API also allows storing the map in the local storage in case the user does not have an Internet connection to load it every time he or she opens the application, the SDK will check if there is a matching map included in the app every time it is requested to load a building. Afterwards, when the building is loaded, the API will keep updating the position of the user while he or she moves. In addition, we will draw a route from the current position, which will be constantly updating, to the destination of the user.

Regarding the Google Glass app, users can launch it in three different ways:

- Static card: when users receive a notification of an emergency, the notification card provides a link to launch the application.
- Voice menu: Google Glass counts with a built-in voice menu that will be displayed if the user says “ok glass”. Inside that menu, our app is shown and is accessible by saying “Evacuation app”.
- Tactile menu: the application is also available on the home screen when the users taps on the touchpad.

The application also allows experimenting with several strategies for a decentralized evacuation support [55] based on different risk avoidance strategies of the users. Users can select to go to the closest exit, to the safest exit (the greatest distance from the emergency) or to the least crowded exit, using the voice interface or the integrated menu system.

**Figure 10:**
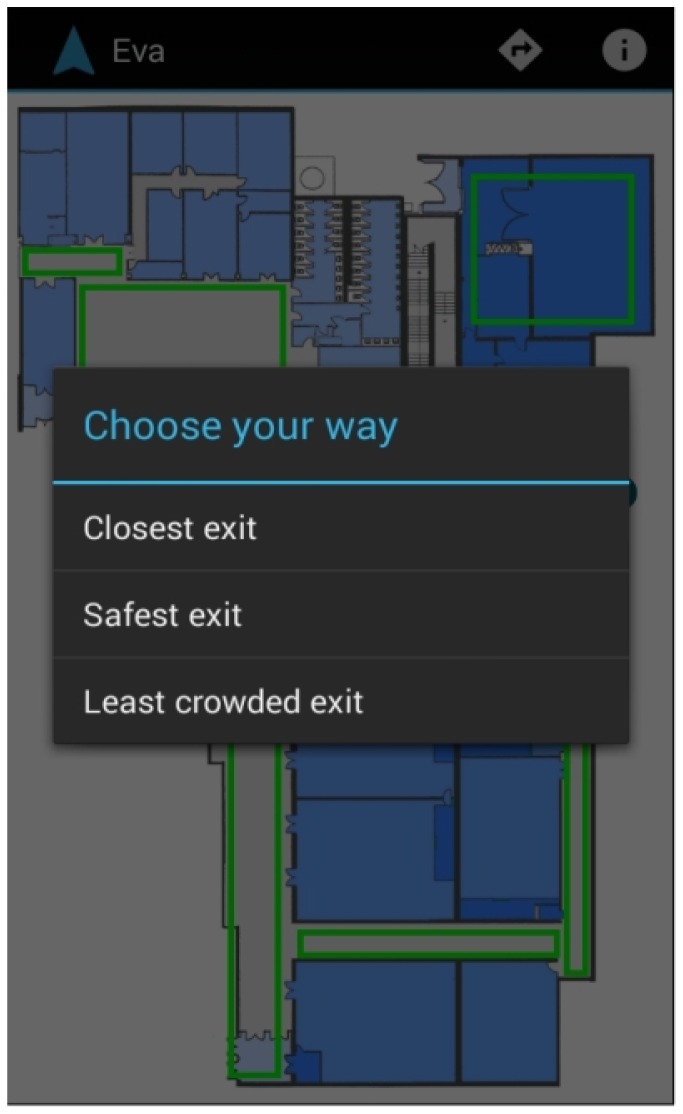
Smartphone interface.

**Figure 11:**
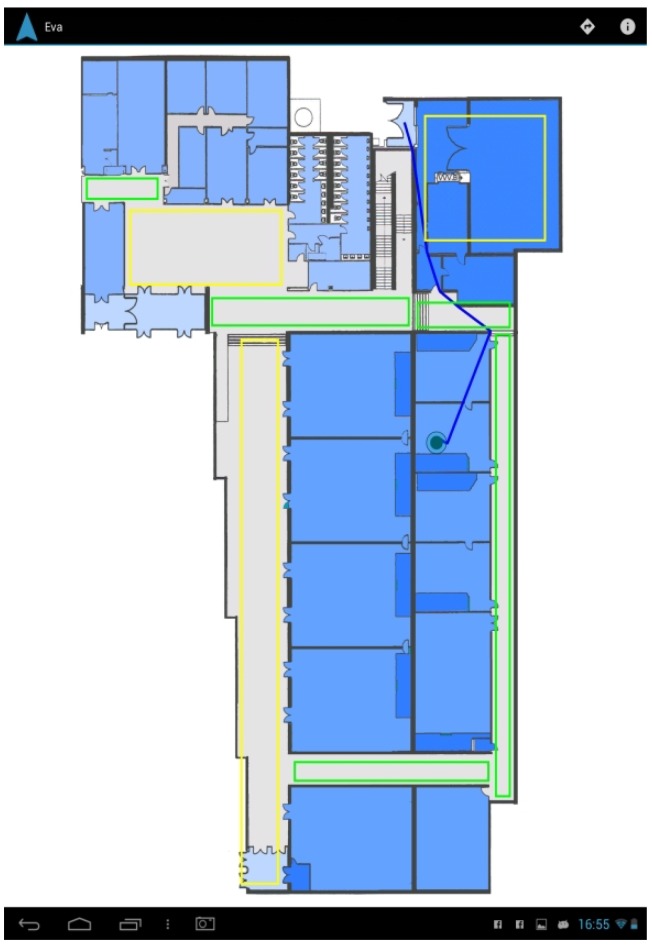
Tablet interface.

The application has been adapted to Android mobile phones and tablets (Figure 10 and Figure 11). This version provides the same functionality except for the specific features of Google Glass, such as voice commands to follow Google’s hands-free philosophy or the tactile menu.

## 5. Experimentation

The system has been evaluated in a real-life scenario, the ground floor of Building B of the School of Telecommunication Engineers (ETSIT) of the Universidad Politécnica de Madrid (UPM).

In order to adapt the system to this scenario, the map of the building has been modeled in UbikSim, and we have used WiFi fingerprinting for indoor positioning, which requires recording the WiFi signal strength in the scenario and uploading to the indoor positioning cloud service (INDOO.RS), so that it can compare later the WiFi received signal strength during the execution of the participatory simulation.

**Figure 12:**
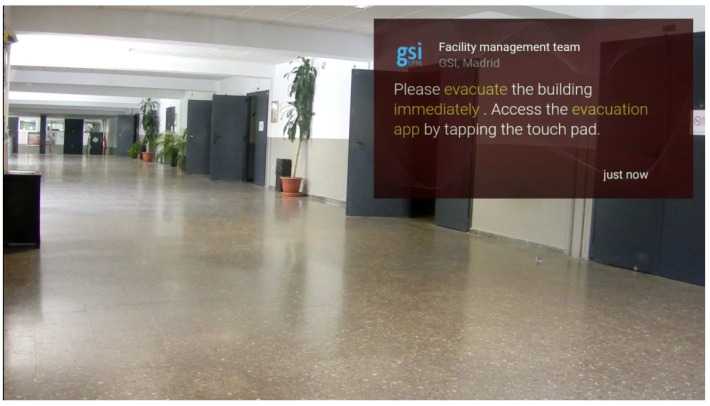
Notification in the Google Glass timeline.

**Figure 13:**
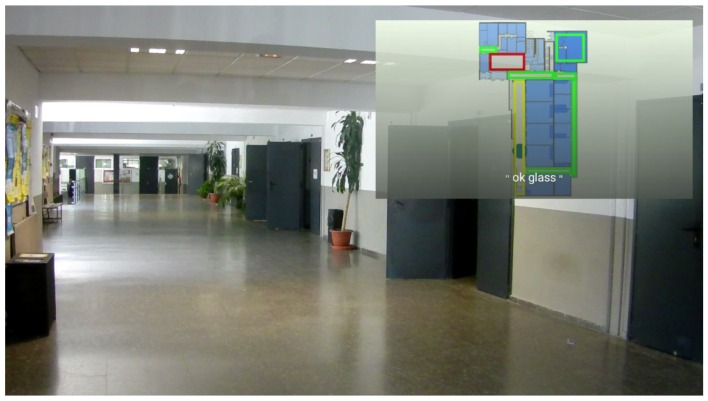
Augmented view in the Google Glass interface.

Based on the indoor localization system, users receive accurate and updated information about their position and evacuation routes, as shown in Figure 12 and Figure 13.

As already stated, the contribution of this paper is proposing an architecture for participatory multi-agent simulation combining smart devices, such as Google Glass or smartphones, with the social engine simulator. An evacuation system has been developed as an application based on the proposed architecture. Thus, we would like to evaluate the developed system testing different hypotheses: (H1) the Google Glass device increases users’ satisfaction, making the usage simpler by letting the user be empty-handed and be focused on the surroundings and not looking down to see the mobile screen; (H2) users’ satisfaction improves when increasing the number of participants involved in the simulation; (H3) the evacuation system modifies people’s behavior during emergencies; and (H4) people follow evacuation indications better in unknown buildings than in well-known places.

### 5.1. Materials and Methods

To test these hypotheses, we conducted an empirical study in which participants interact with the system in both devices (Google Glass and smartphone/tablet), and we evaluate its experience through the process. In order to avoid the effect of possible confounding variable, map representation in both devices is the same, only changing the user interaction due the particularities of each device. To reduce subjectivity, the starting position is the same in all of the experiments.

Participants ranged in age from 20 to 25 years old. All of them were students and knew the building where the participatory simulation took place. They received a brief explanation about the use of the system, specifically the Google Glass device. None of them had participated in any previous study involving evacuation systems.

The 16 participants that volunteered for this evaluation process were randomly assigned to one of two groups to test the first hypothesis. Fifty percent of them tested the system in Google Glass before and then in a smartphone. The remaining 50% used the smartphone first and then Google Glass.

Each participant received a questionnaire at the end of the experiment. Due to some technical restrictions, the maximum number of real people at the same time in the simulation is 5. Therefore, all of the experiments have been done in groups of 5 people or fewer, one with Google Glass and the rest with mobile devices. Each participant is given the freedom to choose the type of exit he or she wants to follow, the default option being to get a route to the safest exit. To test the second hypothesis, we have done the experiments with one person alone, in a group of 3 and in a group of 5.

The evaluation process consisted of two steps. Initially, the participants try to leave the building while simulating the emergency context. Then, they are given a satisfaction questionnaire about the whole experience, as shown in Table 2.

All of the questions have a Likert response bipolar scaling [56] with an interval from 1 to 5, except the one regarding overall satisfactions with the system was 1 to 10 in order to have a better detail in the answers. Number 1 represents the most negative answer and 5 or 10 the most positive one.

### 5.2. Results

**Figure 14:**
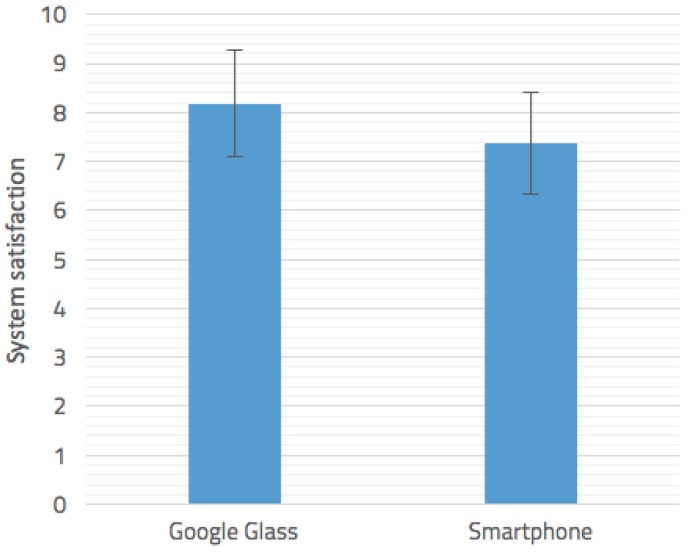
Google Glass vs. smartphones.

Regarding Hypothesis 1, results show that the average satisfaction per user with the two different interfaces was 8.18 (Google Glass) and 7.375 (Mobile device). The mean and standard deviation of the satisfaction with both devices are shown in Figure 14. The ANOVA analysis that we performed indicated that the difference was statistically significant (*p* = 0.0342 < 0.05). No group effects were observed regarding the counterbalancing. These results support our first hypothesis, concluding that users appreciate the Google Glass interface rather than the smartphone one, which show a better acceptance of hands-free devices for emergency situations.

**Figure 15:**
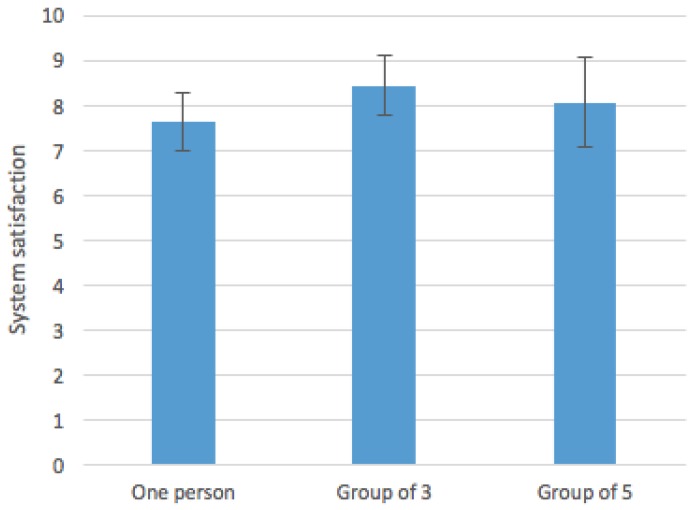
System satisfaction per group.

Regarding Hypothesis 2, we can check that the average satisfaction per user was 7.62 (1 person), 8.43 (group of three people) and 8.06 (group of five). The mean and standard deviation of all of the groups are shown in Figure 15. The performed ANOVA analysis indicated that the difference was statistically significant (*p* = 0.0407 < 0.05). These results also support our second hypothesis, concluding that users have a better experience when being in the simulated evacuation with more people, because the satisfaction of both group options (i.e., three and five people) is above the individual option. Moreover, we can observe a decrease in the average score for the five people option, but the standard deviation increases. This divergence could be due to the usage of the free version of the indoor positioning service INDOO.RS, because it shows some slow response when several users are connected to the application at the same time. That would decrease considerably the satisfaction of the final user. However, as a conclusion, the collected data show an acceptance of participatory simulations to train people for emergency situations showing good satisfaction rates both in groups or individually.

**Figure 16:**
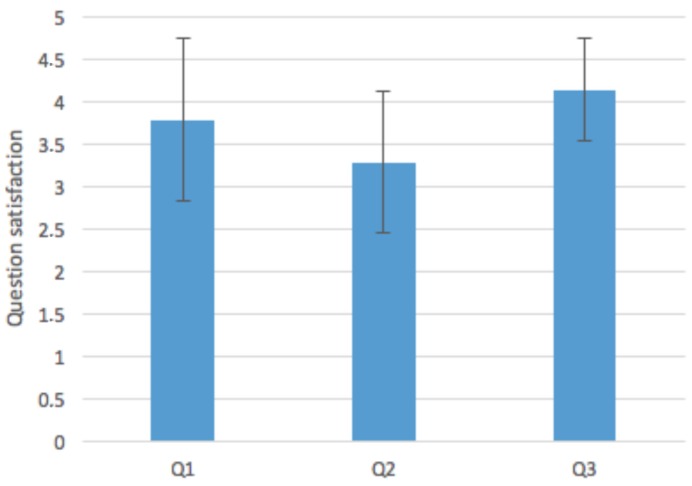
Question satisfaction.

Regarding Hypothesis 3, we can observe that people would have followed the same route in many cases (Q1: AVG= 3.78; SD = 0.95) (results of the questions Q1–Q3 are shown in Table 2, in Figure 16). This fact is understandable because all users that participated in the evaluation knew the building previously. Moreover, they have only an average confidence in this type of system (Q2: AVG = 3.28; SD = 0.83). Thus, we cannot ensure that our system modifies people’s behavior during emergencies with only one evacuation simulation. We would need several training seasons to increase the confidence of the users in the system and to check if their behavior is modified or not. In further development, we plan to improve the interface and user experience by modifying the indoor positioning external service with Bluetooth beacons [57]. This could improve the user experience and, consequently, the confidence in the system.

Finally, regarding Hypothesis 4, the results show that users will follow the evacuation instructions with more confidence if they do not know the building or the space in which they are (Q3: AVG = 4.14; SD = 0.59), which is interesting for using this type of augmented reality systems in public locations, such as stadiums, hospitals or museums. For further iterations, we plan to evaluate our system in complex buildings with users who do not know their structure and analyze how users perceive this type of augmented reality systems for evacuations.

## 6. Conclusions

This paper presents a participatory agent-based simulation architecture for an evacuation scenario, where users can receive evacuation instructions using Google Glass, smartphones and tablets. The article proposes a RESTful architecture for participatory agent-based simulations that has been implemented and evaluated. In this work, we have extended an existing agent-based social simulator UbikSim, which required a low effort thanks to the RESTful architecture. That extension has enabled its integration with smart devices, such as smartphones, tablets or Google Glass, and services, such as the management web application. Moreover, the wide availability of smartphones and tablets makes our system be able to be widely used on the campus. It also allows us to experiment with the use and potential of the wearable device Google Glass, which provides users an augmented view of the real scenario.

During the design phase, we evaluated other possibilities for representing avatars and simulated users, so that the scenario could be more realistic in an augmented reality approach, but we faced restrictions on the capability of Google Glass to process large quantities of information. Thus, we simplified the user interface. With the experimentation phase, we verified a set of hypotheses. Briefly: (i) users prefer to use a hands-free devices, like Google Glass, in evacuation systems; (ii) participants felt more confident when they were with more people in the simulated evacuation than when they used the application individually; that encourage us to continue with the participatory simulation approach to train people for emergency situations; finally, (iii) people perceive this type of evacuation system as useful in unknown buildings with a complex structure.

As future work, we plan to explore different paths once we have tested the first version of the system. We would like to compare egress times with and without the evacuation application and with different devices. Then, we will analyze if the use of a hand-free device, such as Google Glass, reduces the egress time in contrast with other options, such as smartphones or tablets.

Furthermore, we plan to explore the possibility of running the guiding evacuation system in an isolated device without any external connection to the network, which could improve user experience and increase their confidence in the system. This approach would enable the application of the evacuation system, not only for training, but for evacuating people in real emergencies. However, the system would lose real-time information, which is key for efficient evacuation.

Finally, we plan to research shared evacuation plans. That means that the evacuation system would takes into account affinity relationships between evacuees and multiple behaviors, such as hysteria and paralysis. This would enable more realistic scenarios with some crowed exits for hysteric people or the fact that all of the members of a family would follow the same root to escape.

## Figures and Tables

**Table 1:** RESTful interface for resources in the evacuation scenario.

Methods	Resource	Description
GET	/api/v1/agents	List of agents and their positions
GET, PUT, POST, DELETE	/api/v1/agents/<id>	Operations on agent <id>
GET	/api/v1/agents/<id>/routes	Exit routes for agent <id>
GET	/api/v1/rooms	List of rooms
GET, PUT, POST, DELETE	/api/v1/rooms/<id>	Operations on room <id>
GET	/api/v1/locations	Get the list of locations
GET, PUT, POST, DELETE	/api/v1/locations/<id>	Lists
GET	/api/v1/emergencies	List of emergencies
GET, PUT, POST, DELETE	/api/v1/emergencies/<id>	Operations on emergency <id>
PUT	/api/v1/control	Change simulation state

**Table 2:** Questions asked in the experiment.

Hypothesis	Question
3	(Q1) Would you have followed the same way if you had not used the system?
3	(Q2) Do you trust this type of systems, specifically the security part?
4	(Q3) Do you consider this system useful in unknown buildings with a complex structure?
2	(Q4) How many persons were with you in the simulation?
1	(Q5) What type of device did you use?
1,2	(Q6) What is your overall satisfaction about this system?
